# Associations of Serum MicroRNA with Bone Mineral Density in Community-Dwelling Subjects: The Yakumo Study

**DOI:** 10.1155/2020/5047243

**Published:** 2020-07-30

**Authors:** Hiroaki Nakashima, Kei Ando, Kazuyoshi Kobayashi, Taisuke Seki, Shinya Ishizuka, Ryosuke Fujii, Yasuhiko Takegami, Hiroya Yamada, Yoshitaka Ando, Koji Suzuki, Yukiharu Hasegawa, Shiro Imagama

**Affiliations:** ^1^Department of Orthopedic Surgery, Nagoya University Graduate School of Medicine, Aichi, Japan; ^2^Clinical Laboratory Medicine, Fujita Health University Graduate School of Health Sciences, Aichi, Japan; ^3^Department of Rehabilitation, Kansai University of Welfare Science, Osaka, Japan

## Abstract

Osteoporosis is a disease characterized by deterioration of bone tissue and mass, with an increasing global prevalence. Therefore, the discovery of biomarkers for osteoporosis would help to guide appropriate treatment. Circulating microRNAs (miRNAs) have become increasingly recognized as biomarkers for detecting diseases. However, few studies have investigated the association of circulating miRNA with osteoporosis in the general population. The aim of this study was to identify miRNA associated with osteoporosis in a general resident health check-up for potential use as an osteoporosis biomarker. We conducted a cross-sectional study as part of a health check-up program and recruited 352 volunteers (139 men, 213 women, mean age 64.1 ± 9.6 years). Osteoporosis was diagnosed according to the WHO classification. Twenty-two candidate microRNAs were screened through real-time quantitative PCR, and miRNAs associated with osteoporosis were analyzed using logistic regression analysis including other risk factors. In total, 95 females and 30 males were diagnosed with osteoporosis with bone mineral density tests (BMD: *T*‐score < −2.5). We found that miR195 was significantly lower in females, while miR150 and miR222 were significantly higher in males. The results of the logistic regression analysis indicated that in females, higher age and lower miR195 (odds ratio: 0.45, 95% confidential interval: 0.03–0.98) were significant risk factors for lower BMD, while the presence of a smoking habit and lower miR150 (odds ratio: 1.35, 95% confidential interval: 1.02–1.79) were significant risk factors for osteoporosis. Serum levels of miR195 and miR150 are independently associated with low bone mineral density in females and males, respectively.

## 1. Introduction

Osteoporosis is an age-related disease and is the most common bone disease worldwide [1]. Hip or spinal vertebral fractures are frequently observed in patients with osteoporosis, and osteoporotic fractures are one of the leading causes of disability affecting the quality of life in elderly patients [2]. In addition, osteoporotic fractures are increasingly becoming a global problem due to an aging population and longer life span. Thus, it is important to investigate methods for the prevention and treatment of osteoporosis [1, 3]. Osteoporosis often goes undiagnosed until a fracture occurs because patients with osteoporosis are otherwise asymptomatic. If a biomarker for osteoporosis can be identified, it will aid in the diagnosis of osteoporosis and will also provide cell-based information to improve understanding of the pathology of osteoporosis [4].

MicroRNAs (miRNAs), which are short RNAs and subsets of noncoding RNAs of around 22 bases, are candidate biomarkers for osteoporosis. Thousands of miRNAs have been identified in humans and rodents. In general, microRNAs negatively regulate gene expression by binding to the 3′ untranslated region of a specific miRNA through base pairing, thereby suppressing translation and promoting miRNA degradation. In particular, the second to seventh bases at the 5′ end of the miRNA is essential for target miRNA recognition. MicroRNAs may be key molecular regulatory factors in bone metabolism [4]. Given miRNA stability in the blood, circulating miRNAs have been widely reported as potential biomarkers of disease, including cancer, cardiovascular disease, and diabetes [5–7].

Seeliger et al. performed microarray analysis on samples from 10 patients with osteoporotic hip fractures and 10 patients with nonosteoporotic hip fractures. They reported that five miRNAs were upregulated in both the serum and bone tissue of patients with osteoporotic fractures [8]. Several other studies have also reported that specific circulating miRNAs are associated with osteoporosis [9–15]; however, the majority of these studies have used a case-control study design with a small number of cases. No reports appear to have examined the association of circulating miRNA with osteoporotic conditions among community-dwelling subjects in a large cohort. The aim of this study was to identify miRNA associated with osteoporosis in a general resident health check-up for potential use as an osteoporosis biomarker.

## 2. Materials and Methods

### 2.1. Study Subjects

We conducted a cross-sectional study as part of a health check-up program that was conducted in Yakumo Town, Hokkaido, Japan, at the end of August 2012. Yakumo is a relatively rural area, and the main industries here are agriculture and fishery. Yakumo has a population of 18,500 people, of which 27% are elderly (over 65 years old).

Of the 527 residents who attended a health examination in Yakumo, 352 volunteers (139 men, 213 women; mean age: 64.1 ± 9.6 yrs.) underwent a bone mineral density (BMD) and blood sampling test. A self-administered questionnaire was distributed to the applicants before the check-up. All participants in our study provided written, informed consent. Self-reported data were collected, and any missing answers were completed through interviews with the municipal public health nurses at the screening site. The questionnaire was designed to collect data on the individuals' medical history and lifestyle habits, including smoking habits. Body height and weight measurements were obtained to calculate the body mass index (BMI, kg/m^2^).

### 2.2. Bone Mass Density Measurement

A water-bath ultrasound system (model A-1000 Plus II; Lunar, Madison, WI, USA) was used for the measurement of bone status parameters in the calcaneus region of the independent foot [16]. Stiffness (automatically calculated from broadband ultrasound attenuation and sound speed), *T*-score, percentage young adult mean (YAM), *Z*-score, and percentage age-matched value were recorded using a standard protocol provided by the manufacturer. Based on the WHO classification, subjects with *T*-scores of −1.0 and above, between −2.5 and −1.0, and −2.5 and below were considered normal, osteopenia, and osteoporosis, respectively. We compared subjects between normal and osteoporosis in the current study.

### 2.3. Measurement of miRNA

MicroRNAs were analyzed using previously reported methods [17, 18]. In brief, fasting blood samples were taken from subjects, and sera were separated from blood cells within 1 h by centrifugation, then stored at −80°C until analysis. An autoanalyzer (JCS-BM1650, Nihon Denshi Co., Tokyo, Japan) was used for biochemical analysis. TRIzol reagent (Invitrogen, USA) was used to isolate serum miRNAs following the manufacturer's instructions.

The levels of 22 miRNAs in sera (let7d, miR1, miR17, miR20a, miR21, miR27a, miR34a,miR92, miR103a, miR122, miR126, miR130a, miR133a, miR146, miR150, miR192, miR195, miR197, miR199, miR221, miR222, and miR320) were measured using quantitative real-time polymerase chain reaction (PCR) as previously described [17, 18]. These miRNAs are reportedly associated with lifestyle diseases including lipid metabolism, arteriosclerosis, chronic kidney disease, and cardiovascular disease. The miRNAs were used to analyze risk factors for lifestyle-related diseases in previous studies; however, in this study, we used them in osteoporosis risk factor analysis. Serum levels of miRNA expression in subjects with osteoporosis (*T*‐score < −2.5) were calibrated relative to those among subjects with normal *T*-scores (≥−1.0).

### 2.4. Statistical Analysis

Continuous variables are presented as the mean ± standard deviation, and categorical data are presented as percentages. Student's *t*-test was used to compare the continuous variables between groups, and a *χ*^2^ test was used for the categorical data. A logistic regression analysis was performed to investigate risk factors for osteoporosis. Because age and presence of diabetes mellitus were reported as risk factors of osteoporosis, these two factors were also included, with *p* values < 0.20 in univariate and multivariate analyses. A *p* value of <0.05 was considered statistically significant. All analyses were conducted using SPSS version 26 (SPSS, Chicago, IL, USA).

## 3. Results

In total, 95 females (44.6%) and 30 males (21.6%) were diagnosed with osteoporosis (*T*‐score < −2.5) (Table 1). Female subjects with lower *T*-scores (<−2.5) were significantly older (66.6 ± 8.0 vs. 55.4 ± 8.9, *p* < 0.001) and had a higher prevalence of postmenopause (89.8% vs. 60.8%, *p* < 0.001) and hypertension (41.1% vs. 23.5%, *p* = 0.03) compared to subjects with normal *T*-scores (Table 1). Male subjects with lower *T*-scores (<−2.5) displayed significantly higher rates of smoking (86.7% vs. 58.4%, *p* = 0.008) and diabetes mellitus (23.3% vs. 5.7%, *p* = 0.02) compared to subjects with normal *T*-scores. Unlike the female subjects, there was no significant difference in age between males with normal and osteoporotic *T*-scores (Table 1).

**Figure 1:**
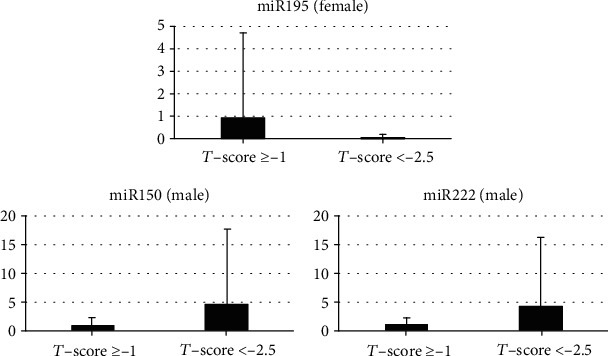
Quantitative real-time RT-PCR analysis of serum levels for five miRNAs (miR-195, miR-150, and miR-222). miR195 levels were significantly lower (0.03 ± 0.2 vs. 1.0 ± 3.7, *p* = 0.01) in female subjects with low *T*-scores, and miR150 (4.6 ± 13.1 vs. 0.03 ± 0.2, *p* = 0.05) and miR222 (4.3 ± 11.9 vs. 1.0 ± 1.2, *p* = 0.05) levels were significantly higher in males.

The miRNA analysis revealed that miR195 levels were significantly lower (0.03 ± 0.2 vs. 1.0 ± 3.7, *p* = 0.01) in female subjects with low *T*-scores (< −2.5) compared to those with normal T-scores (Table 1 and Figure 1). In the male subjects, miR150 (4.6 ± 13.1 vs. 0.03 ± 0.2, *p* = 0.05) and miR222 (4.3 ± 11.9 vs. 1.0 ± 1.2, *p* = 0.05) levels were significantly higher in male subjects with low *T*-scores (<−2.5) compared to those with normal *T*-scores (Table 1 and Figure 1).

The logistic regression analysis indicated that higher age (odds ratio: 1.21, 95% confidence interval: 1.10–1.33, *p* < 0.001) and miR195 (odds ratio: 0.45, 95% confidential interval: 0.03–0.98, *p* = 0.04) were significant risk factors for female subjects with low *T*-scores (<−2.5) (Table 2). In addition, the logistic regression analysis for lower *T*-score (<−2.5) in males revealed that miR150 (odds ratio: 1.35, 95% confidential interval: 1.02–1.79, *p* = 0.04) and the presence of a smoking habit (model 1 including miR150: odds ratio: 2.38, 95% confidential interval: 1.05–5.41, *p* = 0.04; model 2 including miR222: odds ratio: 2.71, 95% confidential interval: 1.24–5.92, *p* = 0.01) were significant risk factors for osteoporosis; however, miR222 was not statistically significant (Table 3).

## 4. Discussion

Several serum miRNAs have been reported to be associated with osteoporosis (Table 4); however, a large-scale cohort study to investigate the relationship between miRNA and osteoporosis is needed. Thus, in our current study, we conducted a resident health check-up on 352 subjects and we identified that miR195 and miR150 were associated with osteoporosis in females and males, respectively. These two miRNAs were reported to have a relationship with bone metabolism in experiments on mice, and these miRNAs could also be potential biomarker for osteoporosis.

miR195 belongs to the miR-15 family, which are induced by stress and activated in several diseases including cancer, cardiovascular diseases, and psychologic diseases [19–21]. miR195 is associated with cell invasion, proliferation, angiogenesis, and apoptosis in multiple cells [20–22], and a relationship with bone metabolism has also been reported. With respect to bone metabolism, miR195 involvement with chondrocyte differentiation and bone morphogenetic protein (BMP) signal has been reported [23, 24]. miR195 inhibits the proliferation and migration of chondrocytes by targeting G-protein-coupled receptor kinase interacting protein-1 (GIT1) [23]. GIT1 is a key regulator of bone mass *in vivo* by regulating osteoclast function [25], and miR195 might affect osteoporosis pathology by controlling chondrocyte differentiation and bone mass regulation. In addition, Grunhagen et al. reported that miR195-5p alters the gene regulatory network of osteoblast differentiation and impairs the induction of BMP responsive genes [24]. Overall, because miR195 is associated with chondrocyte differentiation and bone metabolism in mice, this miRNA is likely to be involved in osteoporosis.

miR150, located in human chromosome 19q13.33, is highly expressed in the lymph node and spleen, playing an important role in immunogenesis, hematopoiesis, and embryogenesis [26]. In particular, miR150 is associated with controlling the maturation of immune cells (B and T cell differentiation, natural killer, and invariant natural killer T cell development) in the bone marrow [26–30]. Therefore, the deregulated expression of miR150 results in the development of autoimmune diseases and hematopoietic malignancies [31, 32]. Although the role of miR150 in osteoporosis has not been reported, Choi et al. investigated the function of miR150 in the bone using miR150 knockout mice. miR150 expression is gradually downregulated through receptor activator of nuclear factor-*κ*B ligand- (RANKL-) mediated differentiation of normal bone marrow-derived macrophages into osteoclasts. miR150 knockout mice had significantly lower serum levels of osteoprotegerin (OPG), but not RANKL, interferon *γ* (IFN-*γ*), or tumor necrosis factor *α* (TNF-*α*) [28]. Furthermore, Dong et al. reported that miR150 affects the osteoblastic phenotype related to osteoblast function and bone mineralization [33]. They reported that increased miR150 levels stimulated osteoblast function and promoted bone mineralization for bone formation in MC3T3-E1 cells [33]. One of potential targets of miR150 is matrix metalloproteinase (MMP) 14 in TargetScan software [33]. MMP14 is associated with cell proliferation [34], so miR150 may affect osteoblast proliferation through the downregulation of MMP14 [33]. In the current study, miR150 levels were approximately five times higher in males with low BMD. This upregulation is difficult to interpret because it is different from the knockdown or downregulation of miR150 in osteoporosis reported in previous studies [28, 33]. However, considering that miR150 is involved in osteoclast and osteoblast activity, it is likely that miR150 has some effect on osteoporosis.

The present study has a couple of limitations. First, we used a quantitative ultrasound (QUS) bone densitometer rather than dual X-ray absorptiometry (DXA) to evaluate osteoporosis. The current study was conducted during a resident medical check-up, so it was not practical to bring in the large device without radiation exposure required for DXA. It has been reported that the *T*-score measured by QUS is strongly associated with BMD measured by DXA [35] but further validation is necessary. Another limitation is that the miRNAs related with osteoporosis selected for this study were measured by quantitative real-time polymerase chain reaction instead of using microarray or RNA sequencing. The strength of the current research is that we investigated miRNAs in a large cohort of subjects but only limited miRNAs was measured by using real-time PCR due to the high cost of microarray or RNA sequencing. Thus, it is necessary to search for a miRNA that is a more effective biomarker for osteoporosis. Our analysis did reveal that miRNA195 and 150 were associated with low BMD, in consensus with previous studies on mice. These results will be useful for better understanding of the pathology of osteoporosis.

## 5. Conclusions

In conclusion, miR195 and 150 are associated with low bone mineral density in females and males and these can be considered biomarkers in analyzed population. Thus, they could be applied as biomarkers in other populations after confirmation through subsequent studies.

## Figures and Tables

**Table 1:** Comparison of characteristics between subjects with bone mineral density T‐scores> -1 (normal) and <−2.5 (osteoporotic).

	*T*‐score ≥ −1	*T*‐score < −2.5	*p*	*T*‐score ≥ −1	*T*‐score < −2.5	*p*
	Female			Male		
Number	51	95		53	30	
Age	55.4 ± 8.9	66.6 ± 8.0	<0.001	64.7 ± 9.7	66.4 ± 8.2	0.45
BMI	22.4 ± 3.5	22.7 ± 3.7	0.61	24.5 ± 3.1	24.0 ± 2.7	0.48
Postmenopause	31 (60.8)	88 (89.8)	<0.001			
Smoking (%)	15 (29.4)	17 (17.9)	0.11	31 (58.4)	26 (86.7)	0.008
HL (%)	9 (17.6)	18 (19.0)	0.85	10 (18.9)	8 (26.7)	0.41
HT (%)	12 (23.5)	39 (41.1)	0.03	22 (41.5)	17 (56.7)	0.18
DM (%)	3 (5.9)	7 (7.4)	0.74	3 (5.7)	7 (23.3)	0.02
Heart ischemia (%)	1 (2.0)	6 (6.3)	0.24	2 (3.8)	3 (10.0)	0.25
Brain ischemia (%)	0 (0)	2 (2.1)	0.30	2 (3.8)	0 (0)	0.28
Liver disease (%)	5 (9.8)	9 (9.5)	0.95	4 (7.5)	3 (10.0)	0.70
CRP	0.06 ± 0.04	0.08 ± 0.25	0.63	0.07 ± 0.09	0.08 ± 0.12	0.69
let7d	1.0 ± 0.1	0.7 ± 1.7	0.46	1.0 ± 5.1	0.7 ± 1.3	0.79
miR1	1.0 ± 0.1	1.3 ± 5.0	0.23	1.0 ± 2.9	1.1 ± 1.8	0.81
miR17	1.0 ± 0.1	0.2 ± 1.7	0.72	1.0 ± 1.4	2.7 ± 7.2	0.10
miR20a	1.0 ± 0.3	0.6 ± 2.4	0.64	1.0 ± 2.1	1.4 ± 3.4	0.49
miR21	1.0 ± 0.1	0.8 ± 1.7	0.25	1.0 ± 3.8	0.6 ± 0.9	0.54
miR27a	1.0 ± 0.1	0.3 ± 0.7	0.72	1.0 ± 3.7	0.8 ± 1.2	0.72
miR34a	1.0 ± 1.9	0.07 ± 0.6	0.54	1.0 ± 5.0	0.2 ± 0.2	0.39
miR92	1.0 ± 0.1	0.05 ± 0.2	0.32	1.0 ± 7.3	0.003 ± 0.01	0.46
miR103a	1.0 ± 0.1	0.5 ± 1.9	0.62	1.0 ± 2.8	0.7 ± 1.0	0.56
miR122	1.0 ± 0.1	0.8 ± 2.5	0.33	1.0 ± 5.7	0.2 ± 0.5	0.46
miR126	1.0 ± 0.1	0.8 ± 1.6	0.58	1.0 ± 3.5	1.2 ± 1.7	0.73
miR130a	1.0 ± 0.1	1.3 ± 5.5	0.19	1.0 ± 4.8	2.5 ± 11.5	0.41
miR133a	1.0 ± 0.1	0.6 ± 2.1	0.35	1.0 ± 4.3	4.1 ± 12.1	0.09
miR146	1.0 ± 0.2	0.9 ± 5.1	0.32	1.0 ± 2.3	7.1 ± 28.2	0.12
miR150	1.0 ± 0.1	1.5 ± 5.8	0.38	1.0 ± 1.2	4.6 ± 13.1	0.05
miR192	1.0 ± 0.1	0.2 ± 0.7	0.91	1.0 ± 5.5	0.5 ± 2.5	0.67
miR195	1.0 ± 3.7	0.03 ± 0.2	0.01	1.0 ± 6.7	0.006 ± 0.03	0.42
miR197	1.0 ± 0.1	0.2 ± 0.5	0.52	1.0 ± 1.4	3.2 ± 10.1	0.12
miR199	1.0 ± 0.1	0.5 ± 1.9	0.55	1.0 ± 4.3	1.0 ± 2.9	0.98
miR221	1.0 ± 0.3	0.5 ± 0.8	0.50	1.0 ± 2.3	1.0 ± 1.1	0.95
miR222	1.0 ± 0.5	0.3 ± 0.7	0.30	1.0 ± 1.2	4.3 ± 11.9	0.05
miR320	1.0 ± 0.1	1.3 ± 2.7	0.38	1.0 ± 1.4	8.0 ± 36.6	0.16

BMI: body mass index; HL: hyperlipidemia; DM: diabetes mellitus; HT: hypertension; CRP: high-sensitivity C-reactive protein.

**Table 2:** Logistic regression analysis for risks of osteoporosis in female patients.

	OR	95% CI	*p*
Age	1.21	1.10–1.33	<0.001
Postmenopause	0.57	0.11-2.92	0.50
DM	0.58	0.12–2.86	0.50
Smoking	1.26	0.45–3.53	0.66
miR195	0.45	0.03–0.98	0.04

OR: odds ratio; CI: confidential interval; DM: diabetes mellitus.

**Table 3:** Logistic regression analysis for risks of osteoporosis in male patients.

	OR	95% CI	*p*
miR150			
Age	1.05	0.97–1.12	0.22
DM	4.19	0.80–22.0	0.09
Smoking	2.38	1.05–5.41	0.04
HT	2.47	0.97–6.28	0.06
miR150	1.35	1.02–1.79	0.04
miR222			
Age	1.03	0.96–1.10	0.38
DM	4.35	0.91–20.94	0.07
Smoking	2.71	1.24–5.92	0.01
HT	1.83	0.79–4.23	0.16
miR222	1.08	0.79–1.50	0.63

OR: odds ratio; CI: confidential interval; DM: diabetes mellitus; HT: hypertension.

**Table 4:** Results of studies on serum miRNAs related to osteoporosis.

Authors	Number (age)	miRNA related to osteoporosis
Feichtinger et al. [14]	36 patients (46.6 ± 13.0 years)	miR-29b-3p, miR-324-3p, and miR-550a-3p
Kocijan et al. [13]	36 patients (46.6 ± 13.0 years) and 39 control subjects (46.6 ± 9.4 years)	miR-155-5p, miR-181c-5p, miR-203a, miR-330-3p, and miR-942-5p,
Ramirez-Salazar et al. [12]	20 patients with fracture, 46 patients with osteoporosis, 28 patients with osteopenia, and 42 control	hsa-miR-885-5p, hsa-miR-17-5p, hsa-miR-1227-3p, hsa-miR-23b-3p, hsa-miR-140-3p, hsa-miR-139-5p, hsa-miR-197-3p
Yavropoulou et al. [11]	35 postmenopausal patients with fracture (71 ± 7 years), 35 postmenopausal patients without fracture (68 ± 7 years), and 30 control subjects (68 ± 5 years)	miR-21-5p, miR-23a, miR-29a-3p, miR-124-3p, and miR-2861
Weilner et al. [10]	19 patients with fracture and 18 control	miR-22-3p, miR-328-3p, and let-7g-5p
Seeliger et al. [8]	10 osteoporotic samples and 10 control	miR-21, miR-23a, miR-24, miR-93, miR-100, miR-122a, miR-124a, miR-125b, and miR-148a
Li et al. [9]	120 postmenopausal women	miR-21 and miR-133a
Ma et al. [36]	21 patients with fracture, 86 cases with osteopenia or osteoporosis, and 14 control	miR-181c-5p, miR-497-5p
The current study	352 subjects (139 men, 213 women; mean age: 64.1 ± 9.6 yrs)	miR-195 (female) and miR-150 (male)

## Data Availability

The datasets analyzed during the current study are available from the corresponding author on reasonable request.
